# The clock gene *Gigantea 1* from *Petunia hybrida* coordinates vegetative growth and inflorescence architecture

**DOI:** 10.1038/s41598-019-57145-9

**Published:** 2020-01-14

**Authors:** Claudio Brandoli, César Petri, Marcos Egea-Cortines, Julia Weiss

**Affiliations:** 1 Genética Molecular, Instituto de Biotecnología Vegetal, Edificio I+D+I, Plaza del Hospital s/n, Universidad Politécnica de Cartagena, 30202 Cartagena, Spain; 2 Instituto de Hortofruticultura Subtropical y Mediterránea-UMA-CSIC, Departamento de Fruticultura Subtropical y Mediterránea, 29750 Algarrobo-costa, Málaga, Spain

**Keywords:** Plant sciences, Plant development, Plant genetics

## Abstract

The gene *GIGANTEA* (*GI*) appeared early in land plants. It is a single copy gene in most plants and is found in two to three copies in Solanaceae. We analyzed the silencing of one *GI* copy, *Petunia hybrida GI1* (*PhGI1*), by hairpin RNAs in Petunia in order to gain knowledge about its range of functions. Decreased transcript levels of *PhGI1* were accompanied also by a reduction of *PhGI2*. They were further associated with increased time period between two consecutive peaks for *PhGI1* and *CHANEL (PhCHL)*, the orthologue of the blue light receptor gene *ZEITLUPE* (*ZTL)*, confirming its role in maintaining circadian rhythmicity. Silenced plants were bigger with modified internode length and increased leaf size while flowering time was not altered. We uncovered a new function for *PhGI1* as silenced plants showed reduction of flower bud number and the appearance of two flower buds in the bifurcation point, were normally one flower bud and the inflorescence meristem separate. Furthermore, one of the flower buds consistently showed premature flower abortion. Flowers that developed fully were significantly smaller as a result of decreased cell size. Even so the circadian pattern of volatile emission was unchanged in the silenced lines, flowers emitted 20% less volatiles on fresh weight basis over 24 hours and showed changes in the scent profile. Our results indicate a novel role of *PhGI1* in the development of reproductive organs in Petunia. *PhGI1* therefore represses growth in vegetative plant parts, maintains the typical cymose inflorescence structure, and inhibits premature flower abortion.

## Introduction

The evolution of land plants has included amongst other adaptations the increase in complexity of the circadian clock. Predictable changes in the environment, as light and temperature, are anticipated by the plant circadian clock, which allows them to adjust their developmental and physiological traits. Most detailed studies on plant circadian clock have been performed in *Arabidopsis thaliana*^1^. The plant circadian clock is based on a set of genes forming several overlapping loops interacting with each other via transcriptional and post-translational activation and repression^2^. Based on the time of the day when the mRNA of the gene shows its expression maximum, the genes included in this oscillator have been classified as the morning loop, midday or core loop and evening loop^3^. In Arabidopsis, *LATE ELONGATED HYPOCOTYL* (*LHY)* and *CIRCADIAN CLOCK ASSOCIATED 1* (*CCA1)*, two MYB transcription factors, form the central circadian oscillator complex, together with *PSEUDO RESPONSE REGULATOR 1* (*PRR1*), better known as *TIMING OF CAB1* (*TOC1*). *PSEUDO-RESPONSE REGULATOR 9* (*PRR9*) and 7 (*PRR7*) form the morning loop genes and the evening complex is formed by the three proteins EARLY FLOWERING 3 and 4 (ELF3 and ELF4) and LUX ARHYTHMO (LUX). These clock genes are interconnected via negative autoregulatory feedback loops, meaning that they reciprocally regulate each other^1,4–6^. Light input is received by *ZEITLUPE (ZTL)*, a gene containing an F-box domain and a blue-light sensing domain, which sustains a normal circadian period through proteasome-dependent degradation of the central clock protein *TOC1*^7^. The stabilization of the ZTL protein in turn is obtained through GIGANTEA (GI), a protein with chaperone activity that facilitates ZTL maturation into an active form^8,9^.

Studies in *Arabidopsis thaliana* and other species revealed that the complex wiring of the oscillator network includes an interplay with hormone signaling^10–12^, cell division and expansion^13,14^, primary metabolism^15^, abiotic stress response^16^, the expression of seed storage proteins^17^, biomass production^18–20^, flower orientation^19^ and flower scent emission^21–24^.

The genetic structure of the circadian clock in the picoeukaryote *Ostreococcus* includes two genes, a *TOC1* and a *LHY* ortholog^25,26^. A protein comprising a LOV domain and a histidine kinase appears to function as an entry for light cues^27^. Relative to these two clock genes, other clock genes such as *GI* appear later in evolution and are present in *Marchantia* but not in *Physcomitrella patens*^28^. The gene *GI* encodes a protein that is not fully characterized. It has important functions in plant development including a conserved role in floral transition in *Marchantia* and *Arabidopsis*^29,30^. It plays a role in control of circadian rhythm in Arabidopsis^31^. Furthermore it coordinates both photoperiod-mediated and independent flowering^32,33^, growth cessation^34^, carbohydrate metabolism^35^, salt tolerance^36^ and cold stress response^37^. *GI* also affects hypocotyl growth in Arabidopsis^38,39^, and this function is related to gibberellin signaling, as SPINDLY (SPY) protein, a negative regulator of gibberellin signaling in Arabidopsis and an inhibitor of hypocotyl elongation, interacts with GI protein^39^. Loss of function of *GI* results in long petioles, tall plant height and many rosette leaves, together with delayed flowering time.

Flower formation in Petunia involves the activity of the flower-meristem-identity genes *PETUNIA FLOWERING GENE* (*PFG*) and *ALF* (*ABERRANT LEAF AND FLOWER)*, the Petunia orthologue of *LEAFY* of Arabidopsis, which induce the floral fate in the lateral shoot meristem^40,41^. The typical determinate inflorescence architecture in Petunia is characterized by a bifurcation of the inflorescence meristem, one terminating into a floral meristem, the other maintaining inflorescence identity and repeating the cymose floral pattern. A few mutants show altered architectures, including *extra petals (exp)*, which forms a single terminal flower^40^ and the mutants *alf* and *double top* (*dot*), a homolog of *UNUSUAL FLORAL ORGANS* (*UFO*) from *Arabidopsis*, where the apical floral meristems convert into inflorescence meristems that do not produce flowers^42^. Overexpression of *DOT* leads to the production of a solitary flower^43^. Another gene that determines Petunia inflorescence architecture is *EVERGREEN (EVG)* involved in the activation of *DOT*, the initiation of the floral identity in the apical meristem as well as lateral inflorescence shoot development^44^. Once the floral program is activated, angiosperm flowers form concentric whorls of organs that include sepals, petals, stamens and carpel and this organ specification relies on the combinatorial genetic function of the organ identity genes according to the Petunia ABCD model^45^.

*GI* generally has remained as a single copy gene in most species. Based on the comparison of the Petunia genomes with other Solanaceae, it has been shown that the circadian clock comprises a different set of genes, including *GI*, which in some cases is duplicated or triplicated. The *GI* gene is present in two copies in *P. axillaris* and three in *P. inflata*. It was hypothesized that some duplicated clock genes may have undergone a subfunctionalization or redeployment^46^.

In this work, we have characterized one of the *GI* orthologues from *Petunia hybrida*, *GI1*, by creating loss of function plants, using hairpin RNA constructs of *PhGI1*. Our results demonstrate novel roles of *GI1* during flowering, consisting in the promotion of flower initiation and flower maturation, the maintenance of cymose inflorescence structure as well as a control over the species-specific VOC profile.

## Results

### Silencing of *PhGI1* has minor effects on clock gene expression and rhythmicity

We have previously shown that *PhGI1* and *PhGI2* have similar expression pattern under a 12:12 Light:Dark photoperiod (12:12 LD)^47^. But we also found that under free running conditions of 12:12 DD, it has a significant change in expression. Thus, we analyzed the expression of *PhGI1* as well as *PhGI2* under long photoperiods of 16:8 LD and continuous darkness.

**Figure 1:**
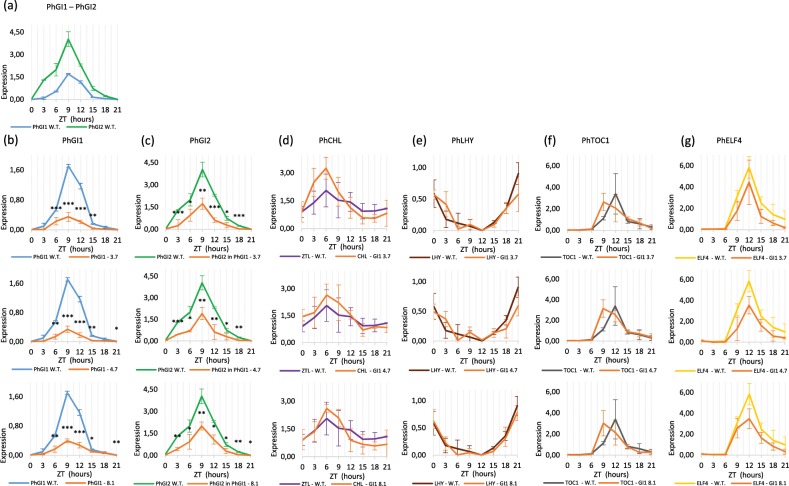
Expression profile during 24 hours of (**a**) *PhGI1* and *PhGI2* in wild type plants and (**b**) PhGI1, (**c**) *PhGI2*, (**d**) *PhCHL*, (**e**) *PhLHY*, (**f**) *PhTOC* and (**g**) *PhELF4* in *iRNA::PhGI1* T1 lines 3.7, 4.7, 8.1 compared to expression in the wild type (from ZT 0 to ZT 15 of light and from ZT 15 to ZT 24 of dark). Expression represents the normalized expression NE according to the formula (NE) = 2^-(Ct experimental – Ct normalization). Three samples were analyzed for each time point and error bars indicate the standard deviation. Asterisks indicate statistical significance between wild type and iRNA lines with *P < 0.05; **P < 0.01; ***P < 0.001 according to Student’s T-test.

The expression pattern of *PhGI1* and *PhGI2* in wildtype leaves is shown in Fig. 1a. Both *PhGI1* and *PhGI2* showed a similar pattern of oscillation with peak expression at 9 hours of light (ZT9). We compared the expression levels of *PhGI1* during 24 hours, measured in 3-hour intervals, and they were inferior to those of *PhGI2* at most time points. Indeed, at ZT9, *PhGI2* expression was double than *PhGI1*. The expression of *PhGI* had been determined previously using an EST from Petunia^24^. A DNA alignment of *PhGI1*, *PhGI2* and the previously reported *PhGI* showed that this EST (FN03636) corresponds to *PhGI2* (Fig. S1).

We silenced *PhGI1* by hairpin RNAi and obtained several independent transgenic lines. We selected three for further work, *iRNA::PhGI1 3.7, 4.7* and *8.1*. As a result of silencing, all *iRNA::PhGI1* lines lacked the strong increase in *PhGI1* expression at ZT9 shown by wild type plants (Fig. 1b) and expression was significantly downregulated at most time points. On average, the peak levels between wild type and iRNA::PhGI lines differed by a factor of 6.

In order to specifically silence only the *PhGI1* without cross-silencing *PhGI2*, we selected a sequence specific for *PhGI*, as indicated by the level of similarity according to the sequence alignment (Fig. S2). However, as shown in Fig. 1c, *PhGI2* was downregulated to 50% in *iRNA::PhGI1* lines compared to the wildtype at peak expression, indicating a certain level of cross-silencing.

Figure 1d–g shows the expression of several circadian genes in the wild type compared to *iRNA::PhGI1* lines. The genes include *CIRCADIAN CLOCK ASSOCIATED1 (LHY) and TIMING OF CAB EXPRESSION1 (TOC1)*, belonging to the core midday loop, and *EARLY FLOWERING 4 (ELF4)* and *CHANEL*, the ortholog of *ZTL* in wild type Petunia, belonging to the evening loop.

In Arabidopsis, *ZTL* messenger RNA is uniformly expressed, but ZTL protein levels oscillate with a threefold change in amplitude. Even so no rhythmic expression of *ZTL* exists in *Arabidopsis* and *Nicotiana attenuata* or *PhCHL*, respectively, in *Petunia hybrida*^21,22,48,49^ a significant rise at ZT9 was observed in Petunia leaves at a 12:12 LD light regime^47^. Similar to this observation we observed peak expression at midday (ZT6), both in wild type and the silenced lines (Fig. 1d).

The expression profile of *PhLHY* (Fig. 1e) over a 24 hour period (16:8 LD) followed the typical peak at the end of the dark period reported for a wide range of tissues in Arabidopsis and other plants^24,50^. This pattern was not altered in the silenced lines of *PhGI1*.

The expression profile of *TOC1* over a 24 hours period (16:8 LD) was characterized by lowest levels during dark period and until midday, followed by an increase to peak expression at 12 hours of light, corresponding to the late afternoon, and a sharp decline towards dark period (Fig. 1f). Peak expression towards the end of the day was also reported for Arabidopsis^51^, soybean^16^ or cowpea^17^. In our case, the expression pattern in *iRNA::PhGI1* lines was similar to wildtype plants however, peak expression was advanced by three hours in all three transgenic silenced lines (Fig. 1f).

The gene *PhELF4*, belonging to the evening loop, showed an identical expression, both concerning pattern and expression level, in wildtype and silenced lines, characterized by a peak expression towards the evening (ZT12), followed by a steady decline towards the end of dark period (Fig. 1g).

The mathematical analysis for circadian oscillation using the JTK_CYCLE algorithm (Table 1) showed that all analyzed clock genes had a rhythmic gene expression pattern, both in wildtype and *iRNA::PhGI1* lines. In case of *GI2, the ZTL-*orthologue *CHL, and LHY*, a significant shift in phase was observed for one or two of the silenced lines, but lacked consistency over all silenced lines. Concerning changes in the time period between two consecutive peaks, a consistent change in all *iRNA::PhGI1* lines was observed for *GI1* and *ZTL-*orthologue *CHL*, which prolonged from 21 to 24 hours.

**Table 1:** Statistical analysis of rhythmicity of gene expression data.

	Pval	Per	Phase	Amp
PhGI1 W.T.	6.98E-11	21	10.5	0.63
PhGI1 3.7	7.00E-08	24	10.5	0.13
PhGI1 4.7	1.48E-08	24	10.5	0.11
PhGI1 8.1	1.48E-06	24	10.5	0.14
PhGI2 W.T.	3.23E-11	24	10.5	1.46
PhGI2 3.7	1.45E-10	24	9	0.47
PhGI2 4.7	2.53E-08	24	9	0.48
PhGI2 8.1	7.61E-10	24	10.5	0.64
CHL W.T.	1.,55E-02	21	7.5	0.47
CHL 3.7	6.96E-07	24	6	1.06
CHL 4.7	1.71E-04	24	7.5	0.72
CHL 8.1	6.88E-05	24	7.5	0.72
LHY W.T.	9.19E-08	24	22.5	0.26
LHY 3.7	1.07E-05	21	1.5	0.23
LHY 4.7	6.32E-06	21	1.5	0.12
LHY 8.1	1.99E-06	24	22.5	0.30
TOC1 W.T.	2.96E-06	24	13.5	0.44
TOC1 3.7	9.11E-06	24	13.5	0.89
TOC1 4.7	9.11E-06	24	13.5	0.89
TOC1 8.1	1.84E-05	24	13.5	0.68
ELF4 W.T.	1.48E-07	24	13.5	1.29
ELF4 3.7	632E-06	24	13.5	0.93
ELF4 4.7	2.96E-06	21	13.5	1.03
ELF4 8.1	1.96E-04	24	13.5	1.50

The P value (Pval) indicates a significative expression rhythm at Pval ≤ 0.05. Period (Per) is defined as the time between two consecutive peaks (expressed in hours). The adjusted phase (Phase), given by JTK_CYCLE and Lomb-Scargle, is considered as the time point with the peak expression (expressed in hours). Amplitude (Amp) is the difference between the peak expression (or minimum expression) and the mean value of the wave.

**Figure 2:**
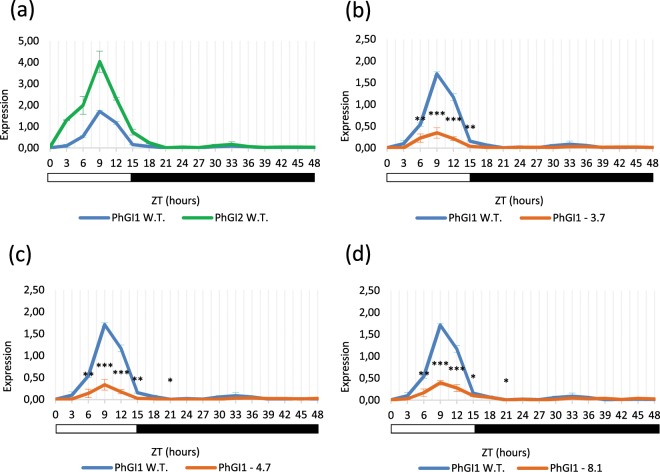
Expression profile in leaves under light and subsequent continuous darkness of (**a**) *PhGI1* and *PhGI2* in wild type plants and *iRNA::PhGI1* T1 lines 3.7 (**b**), 4.7 (**c**) and 8.1 (**d)**. Expression represents the normalized expression NE according to the formula (NE) = 2^-(Ct experimental – Ct normalization). Three samples were analyzed for each time point and error bars indicate the standard deviation. Asterisks indicate statistical significance between wild type and iRNA lines with *P < 0.05; **P < 0.01; ***P < 0.001 according to Student’s T-test.

We analyzed the effect of continuous dark conditions on the expression of *PhGI1* in RNA lines. As shown in Fig. 2, wild type and silenced lines showed the typical high and reduced peak under light towards the afternoon, respectively, but revealed very low basic expression levels during the subsequent continuous darkness. Wild type and silenced lines lost rhythmicity during continuous darkness.

Altogether we can conclude that a strong silencing of *PhGI1* does not have a major effect on the expression pattern or rhythmicity of other clock genes. As GI function is via protein-protein interactions but is not known to be part of a transcriptional complex, it could still have major effects on the protein quantities of PhCHL. Furthermore, the rhythmic expression of *PhGI1* appears to depend on photoperiod.

#### *PhGI1* is a negative regulator of vegetative growth

**Figure 3:**
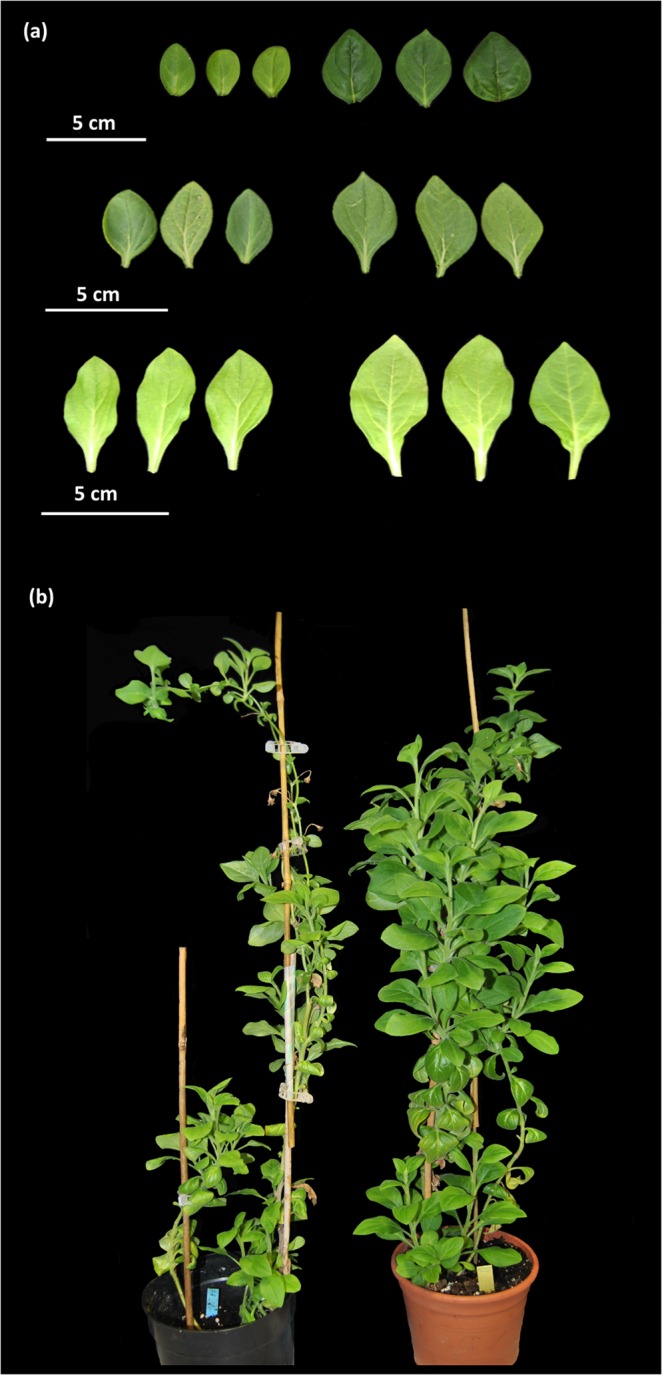
Vegetative growth characteristics in *iRNA::PhGI1* T1 *lines* compared to wild type plants under growth chamber conditions of 16 hours light/ 8 hours darkness. (**a**) From the bottom to the top, basal, medium and apical leaves of three wild type leaves (left) compared to three leaves of *PhGI1* 3.7, 4.7 and 8.1 lines (right) with the strongest silencing. (**b**) Growth habit of the transgenic lines compared to the wild type. Wild type plant (left) and *iRNA::PhGI1* line 4.7 (right).

We analyzed the effect of downregulating *PhGI1* on vegetative growth. The mean value of the vegetative parameters of transgenic plants from T1 and T2 generation, belonging to 6 independent lines (2, 3, 4, 5, 7 and 8), are given in Table 2. Additionally, the results of three different T1 transgenic plants belonging to the three *iRNA::PhGI1* independent lines (3.7, 4.7 and 8.1), are given in Table S1. For the T2 generation we analyzed at least three plants per line. Both generations, grown under long-day conditions, the T1 lines in the growth chamber, and T2 lines in the greenhouse, had significantly longer and broader basal and apical leaves compared to the wild type. Overall, *GI1* silenced plants of the three independent lines showed a remarkable modification in leaf area and growth habit (Fig. 3a,b).

**Table 2:** Comparison of vegetative parameters between wild type and the silenced *PhGI1* in T1 and T2 generation.

Genotype:		W.T.	*iRNA::PhGI1*	% *GI1* versus W.T.	P value
Plant Height (cm)	T1	40.9 ± 0.8	44 ± 4.4	+7.6	2,89E-02
	T2	44.7 ± 6.2	44.6 ± 4.9	−0.2	9,93E-01
Basal Internode (mm)	T1	12.61 ± 0.91	16.93 ± 0.51	+34.4	2,46E-20
	T2	14.28 ± 1.97	17.16 ± 0.79	+20.3	1,40E-03
Median Internode (mm)	T1	16.30 ± 0.56	10.17 ± 0.40	−37.6	3,57E-30
	T2	15.73 ± 1.3	12.44 ± 0.98	−20.8	1,42E-05
Apical Internode (mm)	T1	20.5 ± 1.07	13.60 ± 0.31	−33.7	2,51E-23
	T2	27.9 ± 1.63	23.81 ± 0.82	− 14.6	2,77E-05
N° of leaves to the 1° flower	T1	37 ± 1.4	36.5 ± 1.2	−1.4	7,62E-01
	T2	28.0 ± 1.0	28.7 ± 1.44	+2.5	3,74E-01
N° of axillary meristems	T1	12.5 ± 1.3	27 ± 2.08	+116	3,78E-06
	T2	15.3 ± 1.5	26.6 ± 3.48	+73.9	1,89E-04
N° of branches	T1	2 ± 0.0	2.4 ± 0.57	+20	2,73E-01
	T2	6.7 ± 0.55	6.6 ± 0.7	−1.5	7,80E-01
Basal Leaves length (mm)	T1	64.57 ± 1.04	91.75 ± 5.24	+42	4,42E-33
	T2	90.99 ± 1.55	112.07 ± 6.05	+23.2	3,31E-30
Basal leaves width (mm)	T1	40.93 ± 0.72	50.96 ± 1.53	+24.5	4,97E-30
	T2	49.92 ± 1.6	53.78 ± 1.19	+7.7	4,03E-05
Median Leaves length (mm)	T1	72.72 ± 2.84	75.64 ± 1.66	+4.0	8,23E-02
	T2	65.38 ± 1.74	70.25 ± 1.58	+7.4	2,08E-07
Median leaves width (mm)	T1	43.82 ± 1.68	48.05 ± 1.39	+9.7	1,13E-08
	T2	36.89 ± 1.45	39.84 ± 1.22	+8.0	1,97E-05
Apical leaves length (mm)	T1	38.77 ± 1.31	48 ± 2.91	+24	1,49E-22
	T2	33.15 ± 1.82	36.08 ± 0.81	+8.8	1,36E-03
Apical leaves width (mm)	T1	22.54 ± 0.97	30.53 ± 2.54	+35	2,02E-23
	T2	17.86 ± 0.59	21.96 ± 0.64	+23	2,69E-11
Basal leaves Chlorophyll	T1	22.38 ± 1.09	14.89 ± 1.14	−33.5	1,05E-23
	T2	17.11 ± 1.08	13.84 ± 0.77	−19.1	7,11E-05
Median leaves Chlorophyll	T1	31.04 ± 2.29	37.26 ± 1.07	+20	1,67E-25
	T2	20.14 ±	24.99 ± 0.95	+24.1	2,97E-05
Apical leaves Chlorophyll	T1	21.27 ± 0.95	38.19 ± 1.74	+79.5	2,76E-45
	T2	34.47 ± 0.90	43.57 ± 1.03	+26.4	1,96E-14

Data are given as averages of at least three biological replicates of all silenced plants. The height was calculated from the base to the first flowering meristem. when the first flowering event occurred. The number of total axillary meristems was calculated between the base and the first apical flowering meristem. P values ≤ 0,05 according to Students T-test were considered as significant.

In case of T1 generation, all three lines had a denser apical foliar apparatus, characterized by an apparent increase in the foliar volume. The bushier phenotype may result from an average reduction of the internode length of 37.6% in the median plant region and 33.7% in the apical plant region, while basal internode distance was increased by 34.4%, resulting in overall only slightly taller plants (Table 2). A similar, although minor, significant effect on internode length was observed in the T2 generation, resulting in a plant height identical to wildtype. A marked increase in the number of axillary meristems in both generations may also contribute to a bushier phenotype. However, no significant increases in the number of lateral branches or total leaf number was recorded compared to the wildtype (Table 2).

Transgenic lines had a greener appearance in the denser apical regions, while basal leaves were more yellowish (Fig. 3a). This phenotype coincided with a significant decrease in the relative chlorophyll content in basal leaves and a progressive increase in the median and apical ones compared to the wildtype (Table 2).

We can conclude that *PhGI1* plays a role in vegetative development with a clear acropetal gradient, as it has opposite effects during early stages of development and middle to late stages.

**Figure 4:**
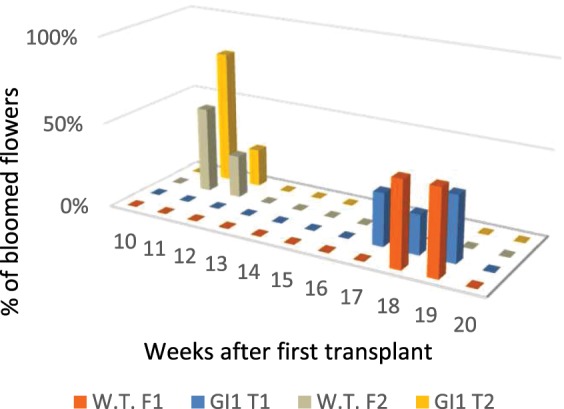
Percentage of fully open flowers in weeks after transplanting from *in vitro* culture to substrate of T1 and T2 generation of *iRNA::PhGI1* lines compared to wild type plant. T1 lines were grown under growth chamber conditions of 16 hours light/ 8 hours darkness. T2 lines were grown in a greenhouse under natural long-day conditions.

Flowering time, expressed as the percentage of plants which fully bloomed after rooted shoots were transferred from *in vitro* jars to pots (in weeks) is given in Fig. 4. *iRNA::PhGI1* lines of T1 and T2 generation and wildtype plants flowered contemporaneously, indicating that in contrast to Arabidopsis, *PhGI1* does not play a role in floral transition. In general, plants kept in the greenhouse flowered five to eight weeks earlier than those kept at 16:8 LD in the growth chamber corroborating that fluence accelerates floral transition in Petunia.

#### *PhGI1* inhibits ectopic flower formation and premature flower senescence

**Figure 5:**
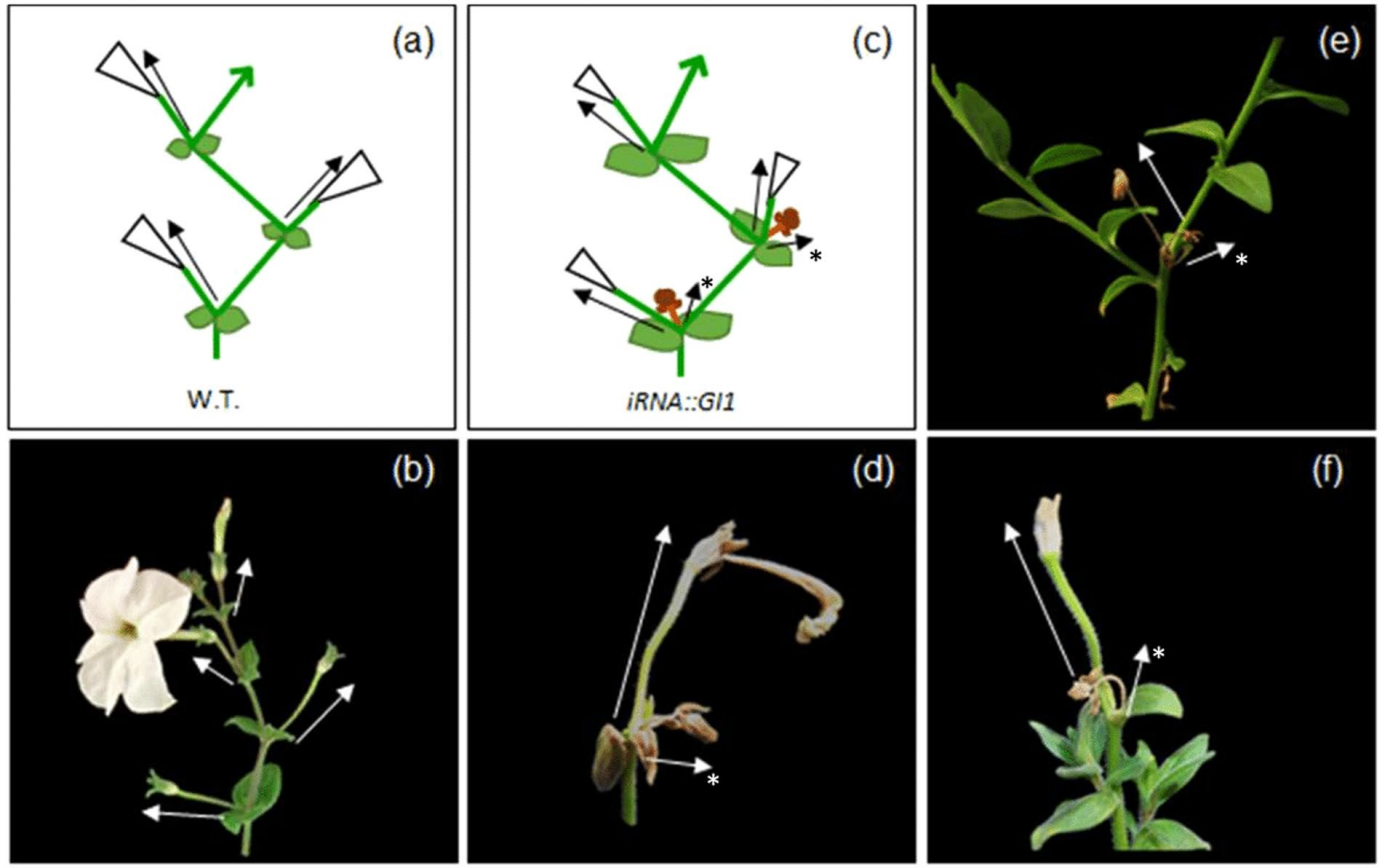
Schematic representation of the petunia inflorescence. Wild type (**a**) and silenced *iRNA::PhGI1* plants of T1 line (**c**). Side view of a wild type petunia inflorescence (**b**) and *iRNA::PhGI1* plants of T1 line (**d–f**). Arrows indicate the position and direction of the main and aborted (*) floral meristems at each bifurcation.

**Figure 6:**
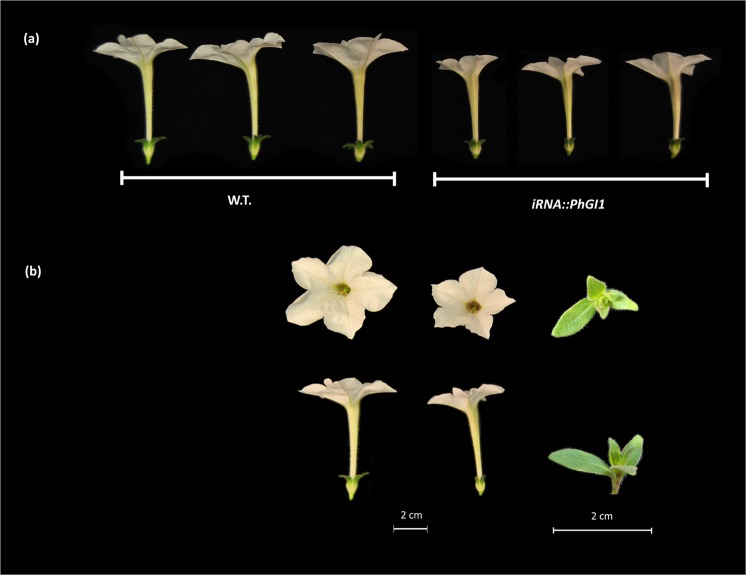
Flower size and flower appearance in T1 lines of *iRNA::PhGI1* compared to the wild type. Tube length (**a**) of the flowers of the wild type (left) and transgenic lines (right). (**b**) Corolla diameter and the abortive flower appearance (extreme right) in T1 lines of *iRNA::PhGI1* (right) compared to the wild type (left).

**Figure 7:**
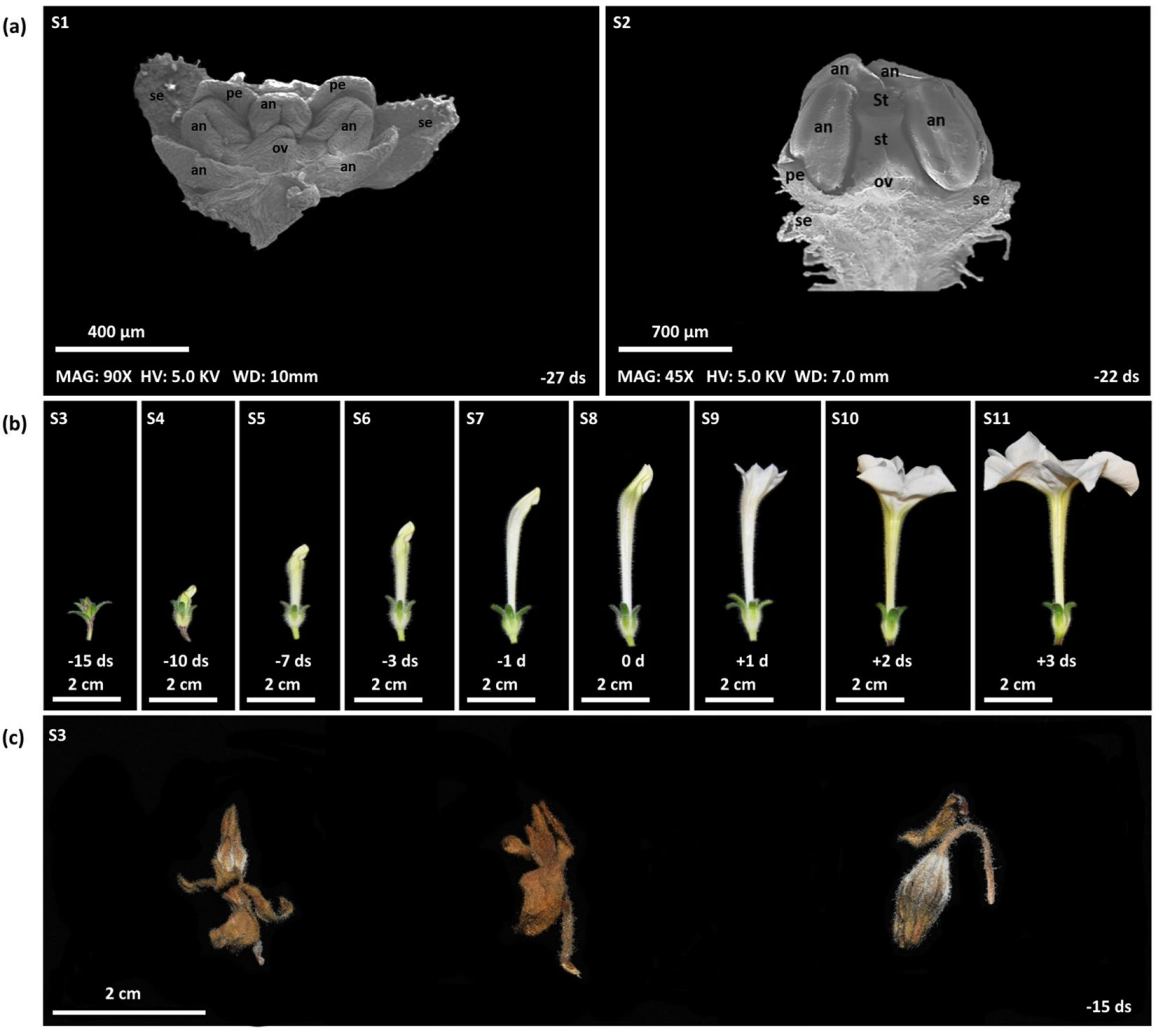
Stages of Petunia flower bud development in wild type and *iRNA::PhGI1* T1 lines. Stages (S) 1–7 represent flower development between 27 to 1 days before anthesis and stages (S) 8–11 represent flowers from 0 to 3 days after anthesis. (**a**) Stages S1 and S2 are given as scanning electron micrographs of the inflorescence apex of Petunia during early stages of development from a wildtype plant (S1) and a plant of *iRNA::PhGI1* T1 line (S2). Stigma (St), style (st), ovary (ov), sepal (se), petal (pe), anthers (an). (**b**) Stages S8–11 of flower buds taken from a position within the plant that develops into a normal flower. (**c**) Stage S3 of flower buds taken from a position within the plant that develops into an aborted flower.

We analyzed floral development in *iRNA::PHGI1* plants compared to the wildtype and observed that even though wildtype plants and silenced plants started to flower concurrently (Fig. 4), the silenced plants developed new inflorescences at a slower pace so that the total number of flower buds at the end of flowering period was reduced by 58 and 59% in the T1 and T2 generation, respectively, compared to the wildtype (Tables 3 and S2). Additionally, we found a striking phenotype that we had not seen previously in wildtype plants. We found that at many bifurcation points were the terminal flower and the inflorescence shoot divide, an additional ectopic flower bud appeared (Fig. 5). While the normally positioned flower bud tended to develop to full maturity, the ectopic flower bud appeared to undergo early senescence and aborted. Ectopic flower buds accounted for 40% in T1 and 21% in T2 lines. As a consequence of ectopic flower bud abortion (Figs. 6 and 7) and slower inflorescence development in the transgenic lines, the final percentage of fully developed flowers diminished to 76% and 67% in the T1 and T2 generation, respectively, compared to wild type plants (Table 3).

**Table 3:** Comparison of floral parameters between wild type and silenced PhGI1 in T1 and T2 generation.

Genotype:		W.T.	*iRNA::PhGI1*	% *GI1* versus W.T.	P value
N° of flower buds	T1	27.8 ± 2.8	11.3 ± 4.1	−59.4	2,21E-05
	T2	29.3 ± 2.3	12.2 ± 5.01	−58.4	2,44E-04
N° of fully developed flowers	T1	27.8 ± 2.8	6.8 ± 2.19	−75.5	1,07E-04
	T2	29.3 ± 2.3	9.6 ± 4.62	−67.2	2,11E-04
% of fully developed flowers	T1	100	60.2	−39.8	1,47E-08
	T2	100	75.0	−25	3,67E-09
Corolla diameter (mm)	T1	46.22 ± 3.41	34.21 ± 2.73	−26.0	4,27E-21
	T2	54.34 ± 3.45	49.66 ± 3.27	−8.6	1,07E-04
Tube length (mm)	T1	40.06 ± 2.10	35.57 ± 2.11	−11.2	2,84E-15
	T2	41.10 ± 1.82	38.79 ± 1.98	−5.6	1,08E-04
Petiole length (mm)	T1	35.96 ± 2.72	35.33 ± 3.20	−1.8	2,67E-01
	T2	47.63 ± 2.51	46.22 ± 3.42	−3.0	5,91E-02

Data are given as averages of at least three biological replicates of all silenced plants. P values ≤ 0,05 according to Students T-test were considered as significant.

The differentiation of stamen and carpel tissue could be clearly observed in the aborting flower buds, even so the pale and brownish coloration in *iRNA* lines indicated an abortion in development, chlorophyll loss and necrosis (Figs. 7 and S3). This early onset of flower senescence occurred well before flowers achieved the normal size of flower opening in Petunia.

**Figure 8:**
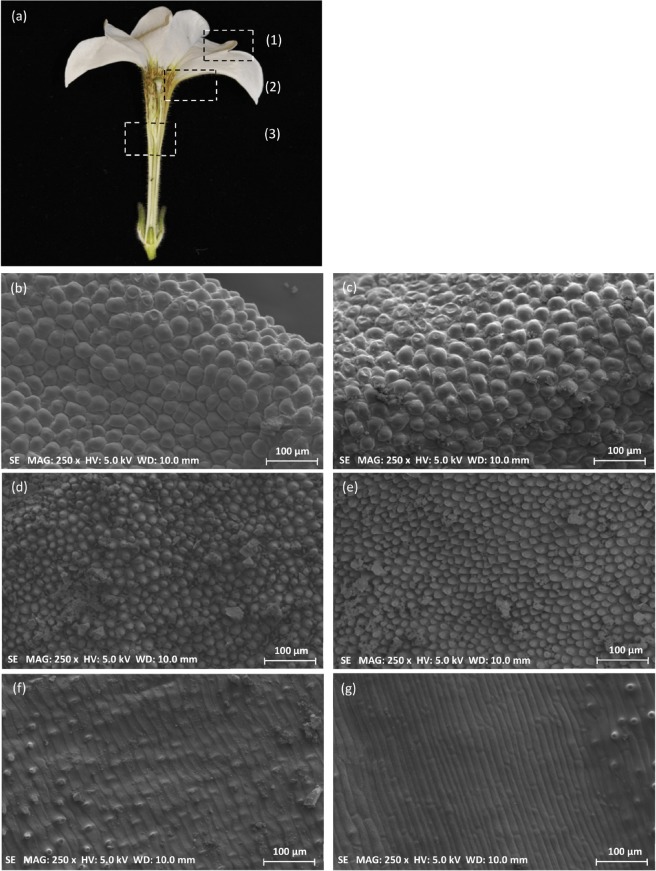
Scanning electron microscopy of petal cell size. Three petal regions were sampled for scanning electroscopic analysis from T1 lines (**a**). Floral cell size comparison between wild type (left) and *iRNA::PhGI1* (right) of different floral organs: (1)(**b,c**) corolla, (2)(**d,e**) limb and (3)(**f,g**) tube.

The flowers of the silenced lines that fully developed appeared to be smaller with a significant reduction both in the corolla diameter as well as the floral tube length (Table 3, Fig. 6). Cell size in the floral tube and two regions of the corolla, the distal outer zone and a proximal zone near the tube, were significantly reduced (Table 4; Fig. 8), indicating an effect of *GI1* silencing over petal cell expansion.

**Table 4:** Comparison of cellular areas of flowers between wild type and silenced PhGI1 plants of T1 generation.

Genotype:	W.T.	*iRNA::PhGI1*	% *GI1* versus W.T.	P value
Corolla (µm^2^)	1132.38 ± 225.13	910.07 ± 171.02	−19.6	8,18E-16
Basal limb (µm^2^)	345.12 ± 88.85	302.07 ± 74.75	−12.5	1,53E-05
Tube (µm^2^)	3653.35 ± 794.4	2566.5 ± 647.14	−29.8	8,81E-28

Values correspond to mean (µm^2^) ± deviation standard error of at least three flowers belonging to the *iRNA::GI1* lines 3.7, 4.7 and 8.1 and 50 measurements for each flower. P values ≤ 0,05 according to Students T-test were considered as significant.

#### *PhGI1* regulates the quantity of volatile emission and fine-tuning of volatile profile

**Figure 9:**
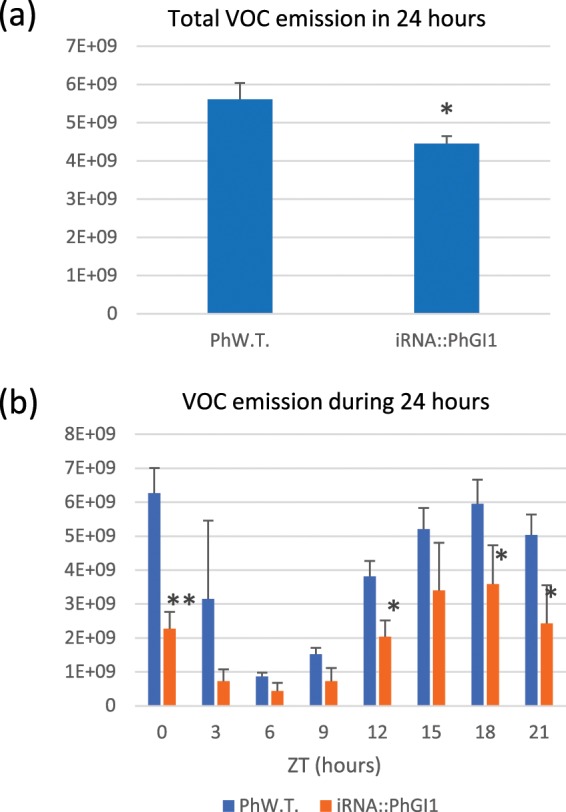
Volatile emission by flowers from wild type and *iRNA::PhGI1* T1 lines. Flowers were excised at ZT0. (**a**) Total VOC emission in wild type flowers compared to *iRNA::PhGI1* lines in 24 hours and **(b**) VOCs emission in three hour intervals during 24 hours. Absolute total emission of VOCs per grams of fresh weight is given as sum of integrated peak area. Asterisks indicate statistical significance between wild type and iRNA lines with *P < 0.05; **P < 0.01; ***P < 0.001 according to Student’s T-test.

**Figure 10:**
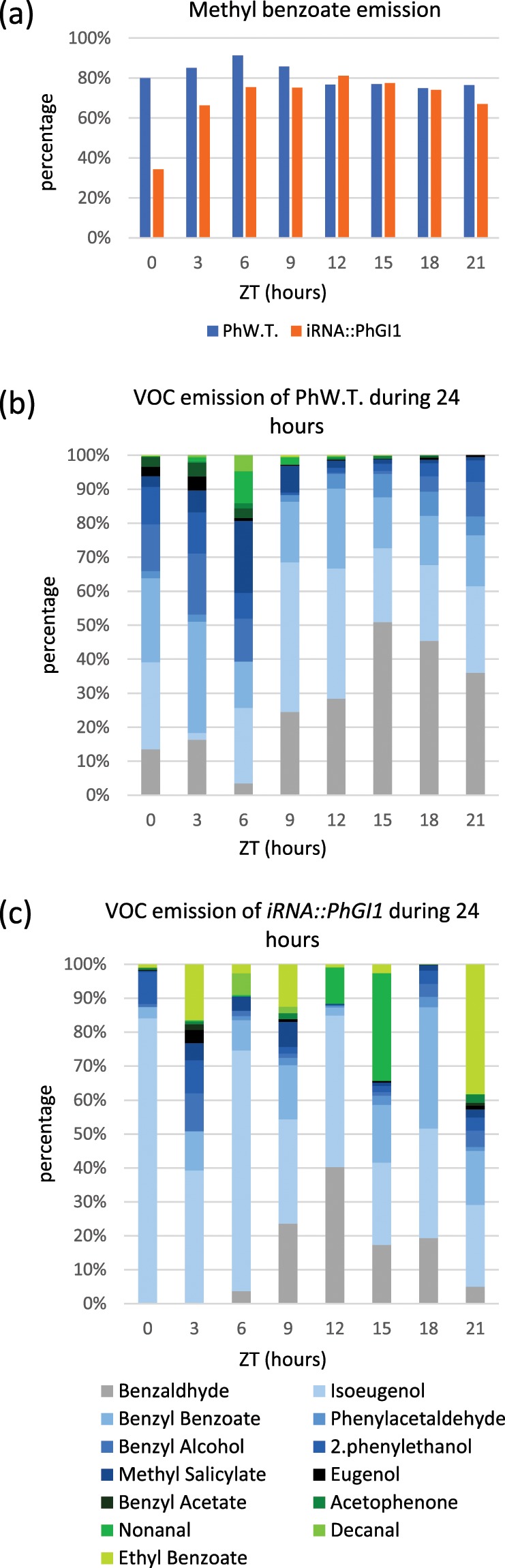
Percent emission of volatile organic compounds (VOCs) from wild type flowers and *iRNA::PhGI1* T1 *lines* 3.7, 4.7 and 8.1 Flowers were excised at ZT0. Methyl benzoate (**a**) and other main VOCs in wildtype flowers (**b**) and *iRNA::PhGI1* lines in three hour intervals during 24 hours. Percentages were calculated based on the integrated peak area divided by flower fresh weight.

The main VOCs were analyzed in the three different plants with the strongest silencing, belonging to three independent *iRNA::PhGI1* lines (3.7, 4.7 and 8.1) and in the wildtype. Data are listed in the Table S3. *iRNA::PhGI1* lines showed an average reduction of 20.6% in total VOC emission on the basis of flower fresh weight in grams (Fig. 9a). Figure 9b shows the rhythm of VOC emission during 24 hours in 3 hour intervals, which was similar in case of wildtype flowers and *iRNA::PhGI1* lines, with lowest emission towards midday at 6 hours of light and increases towards the end of light period with highest emission during the dark phase. We also observed a change in the relative composition (Figs. 10a–c and Table S4). In both, wildtype plants and *iRNA::PhGI1* lines, methyl benzoate was the major volatile with exception of the beginning of light period in *iRNA::PhGI1* lines, when this compound contributed with only 30% to the VOC profile. Concerning the relative contribution of other compounds, we found remarkable changes among wildtype plants and *iRNA::PhGI1* lines, especially with a high contribution of isoeugenol and ethylbenzoate at certain timepoints (Fig. 10b,c). Results indicate that even so the pattern of total emission during 24 hours is quite conserved, individual VOC compounds may change their emission pattern.

## Discussion

In this work we have performed a functional analysis of *GI1* in *Petunia hybrida* by means of loss of function using RNAi lines. There are two paralogs in *Petunia x hybrida* and both *PhGI1* and *PhGI2* showed a pattern of expression in wildtype plants under a 16:8 LD cycle characterized by an increase towards the afternoon at ZT9 followed by a decrease to very low levels during the entire dark period. This pattern is similar to that observed in Arabidopsis, were under long day conditions (16:8 LD), *GI* mRNA peaks at ZT 10 and lowest expression levels occur at ZT 0^30^. Other examples of an evening phased expression pattern for *GI* include cowpea^17,52^ and soybean^16^. In Arabidopsis, CCA1 binds to the *G1* promoter and reduces its expression, which only rises towards midday, when *CCA1* expression is repressed by *TOC1*^53^. We did not observe a robust circadian rhythmicity under continuous darkness for *PhGI1* in the wildtype or the transgenic silenced lines. *PhGI1* expression in Petunia therefore diverges from Arabidopsis, were a strong oscillation of *GI* under conditions of continuous darkness can be observed^54^. Observations on *Petunia hybrida* leaves confirm that *PhGI2* does not maintain rhythmicity during continuous darkness^24^, indicating the necessity of light for the correct expression signals for oscillation of the *GI* paralogs in Petunia.

The specific silencing of a gene for which several paralogs exist within a plant species is very challenging, as it might be difficult to find regions with a sufficient degree of sequence variation. We selected the 3′ untranslated region of *PhGI1* and *PhGI2*, that showed the maximum sequence differences. Even so gene specific sequences were selected for the silencing of *PhGI1* in order to avoid cross-silencing with *PhGI2*, the selected sequence still contained stretches of identical sequences. As a result, we observed a certain level of silencing of *PhGI2*. While *PhGI1* expression levels were down regulated on average 5.6 fold in *iRNA::PhGI1* lines, the reduction of *PhGI2* in these lines was 2.3 fold compared to wild type. Silencing of non-targeted genes was reported to occur if these targets contain as few as eleven contiguous nucleotides of identity with the siRNA sequence^55^, which might explain the observed off-target effects in case of these duplicated *GI* genes. Currently, we cannot determine if the downregulation of *PhGI2* is the result of the *iRNA::PhGI1* construct or if *PhGI1* activates the transcription of *PhGI2* by yet unknown mechanisms, which makes a separation of paralog function difficult.

Expression patterns of the core clock genes *LHY* and *TOC1* were similar in wildtype and silenced lines and similar to Arabidopsis, where *LHY* peaks late during the night and is lowest at the onset of the night while *TOC1* expression is counterphased to *LHY*, forming a negative feedback loop^5^. This feedback control system therefore was not altered by *PhGI1* silencing. Different from Arabidopsis, were *ZTL* messenger RNA is constitutively expressed, we observed a peak expression towards midday of *PhCHL* both in wildtype and silenced lines. A lack of change in *PhCHL* expression in silenced lines can be explained by the fact that in Arabidopsis, interaction of *GI* with *ZTL* occurs at the protein level, consisting in the facilitation of maturation of ZTL into a functional protein. ZTL targets TOC1 for proteasomal degradation^56^, suggesting that changes of TOC1 protein would also be expected. In Arabidopsis, *GI* and *ELF4* have a synergistic effect on endogenous clock regulation, showing epistatic interactions^38^. Furthermore, GI and ELF4 proteins interact physically to form discrete nuclear bodies^57^ but no direct interaction on the expression level is reported, which might explain the similarity in expression pattern between *PhGI1* silenced lines and wildtype.

In petal tissue, silencing of *PhLHY* resulted in a phase-advance of *GI* peak expression of 4 hours^58^, indicating that disturbance of the normal expression pattern of clock genes may alter the rhythmicity of the same and other clock genes. Similarly, the silencing of *GI1* here led to a significant prolongation of rhythmic period of 3 hours for the genes *PhGI1* and *PhCHL* in all silenced lines.

Changes in the vegetative growth of *PhGI1* transgenic lines was characterized by an increased leaf size of basal and apical leaves, and an augmentation in the basal internode length. However, medium and apical internodes were shorter, thus compensating in total plant length, which was not altered. It is well reported that nitrogen concentration diminishes with increasing shoot biomass during plant growth as result of N dilution^59^ and this dilution effect might have contributed to the progressive reduction in internode length in *PhGI1* transgenic lines. Next to the changes in internode length and leaf size, we also observed a structural change in growth characterized by an increased number of axillary meristems as well as a higher chlorophyll level in apical leaves. All changes together led to a bushier phenotype with darker color. In Arabidopsis, *GI* controls the growth of the hypocotyl^38^ and the loss of function of *GI* results, apart from late flowering, in long petioles, tall plant height and many rosette leaves^60^. The findings in Arabidopsis confirm the effect of *GI* on vegetative growth observed in the silenced Petunia lines. *GIGANTEA* is known as a key regulator of flowering time. In Arabidopsis, *GI* mutation leads to a late-flowering phenotype in LD conditions^30^. The role of *GI* in flowering is conferred through its control over *CO* and *FT* mRNA expression levels under inductive conditions as found in different plant species^30^. A second pathway involving *GI* is *CONSTANS* (*CO*) independent and involves *GI* regulation of miR172, which than controls *FT* induction and flowering^32^ We did not observe a switch in inflorescence phase of *iRNA::PhGI1* lines compared to wildtype lines, indicating that *PhGI1* does not share a function in controlling flowering time with *AtGI*. Future research will show whether a case of subfunctionalization has occurred in Petunia were only the second copy of *GI* in petunia, *PhGI2*, affects flowering time. However, the relation between late flowering time and increased biomass seen in Arabidopsis is broken in Petunia as the *PhGI1* silenced lines did not flower later than wild type. In fact, mutant combinations of *RVE* genes in Arabidopsis also disrupt the correlation between biomass production and flowering via changes in *PIF* gene expression^61^. Altogether, our results indicate that *PhGI1* has an effect on plant growth coordination. Interestingly, the analyzed parameters on vegetative and generative growth in general showed a stronger reduction in T1 lines that T2 lines as compared to the wild type. This might be due to the exposure to lower night temperatures of greenhouse grown T2 lines compared to the growth chamber grown T1 lines, as it is known that siRNA generation and silencing is inhibited by low temperatures^62^.

*PhGI1* silenced lines were characterized by a reduction in the number of flower buds, the appearance of two flower buds at the bifurcation point of the inflorescence meristem, of which one flower bud aborted, and an increased overall incidence of premature failure in floral development. All these phenomena were not described until now for any other *GI* mutant. The appearance of ectopic flower buds in the bifurcation point of the inflorescence meristem was not described for any other mutant in *Petunia hybrida*. Mutants affecting flower bud appearance described so far are *double top* (*dot)* and *aberrant flower* (*alf)*, characterized by a failure to develop flowers and *extra petals (exp)* and *evergreen (evg)*, were the inflorescence forms a solitary flower^63,64^. Aborted flowers clearly show carpel and stamen tissues indicating that flower abortion occurred following the activation of genes specifying floral organ identity. On the other hand, the overall reduced number of flower buds suggests an effect of *PhGI1* silencing on upstream events, possibly related the flower-meristem-identity genes *PETUNIA FLOWERING GENE (PFG)* and *ALF* (*ABERRANT LEAF AND FLOWER)*^41,63^. Mutants showing a developmental arrest in flower bud development all belong to the group of gibberellin deficient mutants, including *gibberellin deficient (ga-2*)^65^ and *gib-1*^66^ from tomato or *ga1-1* from Arabidopsis^67^. The promotion of petal, stamen and anther development in Arabidopsis was proposed to occur by opposing the action of the DELLA proteins RGA, RGL1 and RGL2^68^. As mentioned above, *GI* is a negative regulator of growth, as *GI* loss of function mutants show taller plant height^60^ and longer hypocotyls. However, the function of *GI* in flower development seems inverse, as flowers either aborted or showed a reduction in corolla and tube size. The reduced size was accompanied by a significant reduction in cell size, indicating that flower size changes are, at least in part, due to a reduced cell expansion, even so we cannot rule out a possible effect over cell division. Growth of lateral organs starts with cell division, followed by cell expansion during later stages of development^69–72^. Our results indicate that *PhGI1* function on lateral organ growth depends on the acquired meristem identity and that the growth promoting function of *PhGI1* during flower development is restricted rather to developmental stages following organ differentiation, when growth relies on cell expansion.

The floral fragrance in *Petunia hybrida* is dominated by volatile benzenoids, which mostly derive from *trans*-cinnamic acid, whose precursor is phenylalanine. The production of phenylalanine is controlled by *ODORANT1* (*ODO1*), a key volatile regulator and member of the R2R3-type *MYB* family, which controls the synthesis of precursors of the shikimate pathway^73^. The main volatile, methyl benzoate, has its maximum emission at night^74^. It is produced from benzoic acid, whose synthesis might be controlled by PAL^75^. Wildtype Petunia observed here showed a rhythmic emission pattern with maximal emission during the night and methyl benzoate continuously was the major compound throughout the day. Differences between the wildtype and the silenced lines consisted (1) in a lower emission level, (2) in slight changes in the relative abundance of the *trans*-cinnamic acid derivatives benzyl alcohol, ethyl benzoate and benzyl benzoate and (3) a mayor contribution of isoeugenol to the volatile profile in the morning. Isoeugenol also derives from phenylalanine, but its direct precursor was suggested to be ferrulic acid, produced from *trans*-cinnamic acid through coumaric acid and caffeic acid. Our finding suggests that *GI* interacts in the rhythmic fine tuning of volatile biosynthesis and the daily emission profile of volatiles derived through the phenylalanine pathway. We cannot exclude that some changes in emission quantity and quality shortly after sampling might be related to wounding, as it was shown that stress conditions and membrane damage may affect VOC generation^76^.

While the plant circadian clock coordinates environmental inputs into basic processes such as primary and secondary metabolism, cell division or cell expansion, in this work we uncover undescribed functions of *PhGI1* on overall inflorescence architecture. It remains to be determined if the phenotypes found in this study are directly controlled by the clock or are specific functions resulting from neofunctionalization of *GI* genes in Petunia.

## Methods

### Plant material, growth conditions and sampling

Wild type *Petunia hybrida* plants of the double haploid variety ‘Mitchell W115’ as well as silenced lines of the T1 generation of *PhGI1* and their non-transgenic siblings were cultured using a commercial substrate (Universal Substrate, Floragard Betriebs GmbH, Oldenburg, Germany) in a growth chamber under conditions of 16 hours light/ 8 hours darkness, a light intensity of 250 μE m^−2^ s^−1^, and a constant temperature of 26 ± 1 °C. A T2 generation of *PhG*I1 was grown in a greenhouse under natural long-day conditions. Plants were watered as required and transplanted to fresh substrate twice during the growth phase.

Phenotyping of vegetative and generative traits, including the size of three leaves and flowers, internode length, flower number, flowering time and relative chlorophyll content was performed. Parameters were evaluated from three wild type plants and 2–3 plants of each *iRNA::PhGI1* line. Of each autopollinated T1 plant, T2 plants were propagated, of which were characterized at least three plants per silenced line, in order to confirm the RNA interference associated phenotypes.

For *PhGI1* expression analysis, as well as other circadian rhythm related genes, three samples of young leaves, from each of the three independent *iRNA::PhGI1* transgenic lines as well as wildtype plants, were sampled under the aforementioned growth chamber conditions. Tissue sampling was performed every three hours. For the analysis of the expression under continuous darkness, plants were initially acclimated during 4–5 days to conditions of 16 hours of light / 8 hours of darkness, after which we proceeded to keep the plants in continuous darkness for 24 consecutive hours. The collected tissues were immediately frozen in liquid nitrogen and stored at −80 °C until further analysis. To measure the progress of time in hours, we used the ZEITGEBER time scale. The term ZEITGEBER, from the German “time giver”, is often used to indicate an external environmental factor capable of synchronizing the biological clock of an organism. We considered ZEITGEBER 0 (ZT0) as the time lights were turned on.

For the analysis of VOC profiles, we sampled three flowers per plant at 2–3 days after flower opening at ZT0. The measurement of VOC emission was performed as described previously^77,78^. Briefly, flowers were placed in a glass beaker with a solution of 4% of glucose inside a desiccator and emitted volatiles were collected using the SPME methods from the headspace during 24 hours as well as every three hours during 24 hours under 16 h/8 h photoperiod, followed by GC/MS. Volatiles were expressed as integrated peak area divided by flower fresh weight^79^. VOCs that contributed with at least 2% to the total emission are considered as main VOCs.

### Silencing of PhGI1: Generation of vector constructs and transformation

For vector construction, we selected a fragment of the 3′ untranslated region of *PhGI1* that would discriminate between *PhGI1* and *PhGI2*. The sequence information for the comparison between *PhGI1* and *PhGI2* was obtained from the genomic clones *PhGI1* (Peaxi132Scf1428Ctg026) and *PhGI2* (Peaxi132Scf1428Ctg060) identified in *P. hybrida* W115 (Fig. S1). Based on this comparison, we selected a DNA fragment of 225 bp from *PhGI1*, that showed maximal sequence difference with *PhGI2* (Fig. S2) and this fragment was PCR-amplified using site-specific primers containing the attB1 and attB2 sites for Gateway recombination^80^. Genomic DNA was used as template for all fragment amplification. Each fragment was first recombined into the entry vector pDONR201 (Invitrogen) and then recombined into the final destination vector pHELLSGATE12 in order to obtain hairpin-like structures. All primers used for plasmids generation are listed in Table S5.

The W115 Mitchell double haploid was transformed as described before^81^ using *Agrobacterium tumefaciens* strain EHA105. Shoots, developed under selective conditions, were confirmed as transformed through PCR detection of the selection marker gene *nptII* (T0, T1) and DNA blot analysis (T0) with a *npt*II DIG-labeled DNA probe (Fig. S4)^82^.

### Circadian gene expression analysis

Total RNA from leaves was isolated using a phenol:chloroform based protocol^83^. Following spectrophotometric quantification (NanoDrop2000), equal amounts of RNA were used to synthesize cDNA according to the manufacturer’s instructions (Maxima First Strand cDNA Synthesis Kit for RT-qPCR, with dsDNase Thermofisher (https://www.thermofischer.com/, catalog number: K1641).

The gene *ACTIN 11 (ACT)*, previously selected as valuable housekeeping gene for Petunia leaves and petals under circadian conditions^47^ was used as reference gene for relative expression quantification of clock genes. Primers for *PhGI1*, *PhGI2* and other clock genes (Table S5) were designed using pcrEfficiency software^84^. Quantitative PCR and melting point analysis were performed as described previously^22^. Three biological and two technical replicas were analyzed for each sample.

### Chlorophyll content

Chlorophyll content was determined in basal, medium and apical leaves of wildtype plants and three silenced lines of *PhGI1* from T1 generation kept under 16:8 LD light regime. Relative chlorophyll content was calculated using a CM-500 Chlorophyll Meter (SolfrancTecnologías SL) based on measuring light penetration coefficient in a two wavelength range corresponding to red light and IR light.

### Scanning electron microscopy analysis

We observed petal cell size in the corolla and the floral tube of silenced lines from T1 generation and non-transgenic siblings. The two areas were separated with a scalpel blade. From the corolla, we prepared two zones for further analysis of cell size, the distal outer zone and a proximal zone near the tube. Petal sections had a size of approximately 0.75 cm^2^. Cell size was calculated measuring the area of 50 cells from 3 different flowers of 3 plants by using the program ImageJ (ttps://imagej.nih.gov/ij/download.html).

The floral meristems were sampled from flowers of *GI1* silenced line from both positions, those that develop into mature flowers and those that develop into aborted flowers. Preparation of flower buds for scanning electron microscopy consisted in the removal of the sepals. All tissues were dehydrated as previously described^85^, followed by critical point drying.

### Data analysis procedures

Expression of circadian genes relative to the reference genes was analyzed applying the comparative CT method^86,87^ as well as using group-wise comparison with the REST Program^88^. The JTK-Cycle algorithm from the MetaCycle R package (R version 3.3.2)^88,89^ was applied in order to detect rhythmicity in gene expression. Significance differences among data were determined based on Fisher´s F-test and Student´s T-Test after testing data for non-normal distributions.

### Significance statement

The Gigantea gene appeared in land plants and is considered as an activator of floral transition. In Petunia it has several functions including repression of vegetative biomass accumulation, and ectopic flower initiation. In *PhGI1*-silenced plants, flowers either aborted or grew to small sizes, emitting low quantities of scent. *PhGI1* thus shows a new set of functions during flower development.

## Supplementary information

Supplementary information.
