# Supplementary material for: The clock gene Gigantea 1 from Petunia hybrida coordinates vegetative growth and inflorescence architecture
Source: Sci Rep. 2020 Jan 14;10:275. doi: 10.1038/s41598-019-57145-9 (PMC6959227; doi:10.1038/s41598-019-57145-9)
Supplement: Supplementary file 1 — Supplementary information. [file 41598_2019_57145_MOESM1_ESM.pdf]

Supplementary Material

The clock gene *Gigantea 1* from *Petunia hybrida* coordinates vegetative growth and inflorescence architecture

Claudio Brandoli[1], César Petri[2], Marcos Egea-Cortines[1] and Julia Weiss[1*]

Genotype:	W.T.	*iRNA::PhG I1*	*iRNA::PhG I1 (3.7)*	*iRNA::PhG I1 (4.7)*	*iRNA::PhG I1 (8.1)*	% GI1 versus W.T	P value	% GI1 (3.7) versus W.T.	P value	% GI1 (4.7) versus W.T	P value	% GI1 (8.1) versus W.T	P value
**Plant Height (cm)**	40.9 ± 0.8	44 ± 3.5	50	46	43	+7.6	2.89E-02	+22.2		+12.5		+5.1	
**N° of leaves to the 1° flower**	37 ± 1.4	36.5 ± 1.2	30	32	32	-1.4	7.62E-01	-18.9		-13.5		13.5	
**Basal Internode (mm)**	12.6 ± 0.9	16.9 ± 0.5	17.4 ± 0.5	16.7 ± 1.2	16.9 ± 0.7	+34.4	2.46E-20	+38.1 ***	7.64E-05	+40.5 *	2.55E-02	+34.1 **	2.31E-03
**Median Internode (mm)**	16.3 ± 0.6	10.2 ± 0.4	9.6 ± 0.5	9.9 ± 0.2	9.8 ± 0.6	-37.6	3.57E-30	-41.1 ***	2.22E-04	-39.3 ***	4.94E-18	-39.9 ***	7.31E-04
**Apical Internode (mm)**	20.5 ± 1.1	13.6 ± 0.3	13.8 ± 0.8	13.8 ± 0.8	13.4 ± 0.9	-33.7	2.51E-23	-32.7 ***	1.23E-03	-34.1 ***	9.79E-05	-34.6 **	1.96E-03
**N° of axillary meristems**	12.5 ± 1.3	27 ± 2.08	28	29	25	+116.0	4.08E-07	+124		+132		+100	
**N° of branches**	2 ± 0.0	2.4 ± 0.57	2	3	2	+20	2,73E-01	+0		+50		+0	
**Basal Leaves length (mm)**	64.57	91.75	88.2 ± 0.9	92.5 ± 1.5	88.3 ± 1.27	+42.1	4.42E-33	+36.6	1.02E-05	+43.3	2.17E-04	+36.8	1.17E-04
**Basal leaves width (mm)**	40.93	50.96	50.1 ± 0.7	53.8 ± 1.3	44.6 ± 0.5	+24.5	4.97E-30	+22.4	1.98E-05	+31.4	9.49E-04	+9.0	5.61E-07
**Median Leaves length (mm)**	72.7 ± 2.8	75.6 ± 1.6	70.3 ± 1.2	82 ± 1.6	73.4 ± 1.8	+4	8.23E-02	-3.3	5.71E-02	+12.8	2.95E-04	+0.9	6.55E-04
**Median leaves width (mm)**	43.8 ± 1.7	48.1 ± 1.4	45.4 ± 0.1	50.7 ± 1.7	49.4 ± 0.7	+9.7	1.13E-08	+3.6	1.40E-01	+5.5	8.70E-03	+2.1	4.94E-05

**Apical leaves length (mm)**	38.8 ± 1.3	48 ± 2.91	44.9 ± 1.3	49.5 ± 1.5	45.6 ± 1.8	+24	1.49E-22	+16	1.08E-02	+28	1.65E-03	+17.6	1.66E-02
**Apical leaves width (mm)**	22.5 ± 0.9	30.5 ± 2.5	30.1 ± 0.8	28.6 ± 1.6	31.1 ± 1.03	+35	2.02E-23	+34	3.60E-04	+27	1.60E-02	+38	1.65E-03
**Basal leaves Chlorophyll**	22.4 ± 1.1	14.9 ± 1.1	12.00 ± 1.1	13.97 ± 1.6	15.30 ± 0.8	-33.5	1.05E-23	-46.38	7.96E-04	-37.58	1.31E-02	-31.64	1.79E-04
**Median leaves Chlorophyll**	31.04 ± 2.3	37.26 ± 1.1	36.30 ± 0.6	35.97 ± 1.3	39.47 ± 1.4	+20	1.67E-25	+16.95	4.57E-04	+15.88	4.85E-03	+27.16	1.18E-02
**Apical leaves Chlorophyll**	21.27 ± 0.9	38.19 ± 1.7	35.33 ± 1.3	36.17 ± 1.5	38.97 ± 0.9	+79.5	2.76E-45	+66.1	1.26E-03	+70.05	1.29E-03	+83.22	1.58E-04

Table S1. Comparison of vegetative parameters between wild type and the silenced lines 3.7. 4.7 and 8.1 of PhGI1 in the T1 generation. Data are given as averages. based on at least three biological replicates. The height was calculated from the base to the first flowering meristem. when the first flowering event occurs. The number of total axillary meristems was calculated between the base and the first apical flowering meristem. P values ≤ 0.05 according to Students T-test were considered as significant.

Genotype:	W.T.	*iRNA::PhGI 1*	*iRNA::PhGI 1 (3.7)*	*iRNA::PhGI 1 (4.7)*	*iRNA::PhGI 1 (8.1)*	% GI1 versus W.T	P value	% GI1 (3.7) versus W.T.	P value	% GI1 (4.7) versus W.T	P value	% GI1 (8.1) versus W.T	P value
**N° of flower buds**	27.8 ± 2.8	11.3 ± 4.1	18	13	16	-42		-59.4		-35		-53	
**N° of fully developed flowers**	27.8 ± 2.8	6.8 ± 2.2	8	6	9	-68		-75.5		-71		-78	
**% of fully developed flowers**	100	60.2	44	46	56	-44		-39.8		-56		-54	
**Corolla diameter (mm)**	46.22 ± 3.4	34.21 ± 2.7	34.18 ± 2.7	34.38 ± 2.3	35.54 ± 0.7	-23.1	4.27E-21	-26.0	1.14E-04	-26.0	2.24E-05	-25.6	2.36E-15
**Tube length (mm)**	40.06 ± 2.1	35.57 ± 2.1	35.38 ± 2.4	35.86 ± 1.5	35.70 ± 1.9	-10.9	2.84E-15	-11.2	1.59E-03	-11.7	1.09E-03	-10.5	3.53E-03
**Petiole length (mm)**	35.96 ± 2.7	35.33 ± 3.2	36.99 ± 2.9	36.04 ± 2.7	33.83 ± 4.2	-5.9	2.67E-01	-1.8	6.67E-01	-2.9	8.03E-01	-0.2	3.04E-01

Table S2. Comparison of floral parameters between wild type and silenced *PhGI1* in T1 generation. Data are given as averages. based on at least three biological replicates. P values ≤ 0.05 according to Students T-test were considered as significant.

RT (min)	Hit Name	CAS Number
7.318	*Methyl Benzoate*	93-58-3
4.82	*Benzaldhyde*	100-52-7
13.311	*Isoeugenol* (isomers)	97-45-1; 5932-68-3
17.44	*Benzyl Benzoate*	120-51-4
6.711	*Phenylacetaldehyde*	122-78-1
6.511	*Benzyl Alcohol*	100-51-6
8.111	*2.phenylethanol*	60-12-8
9.615	*Methyl Salicylate*	119-36-8
12.211	*Eugenol*	97-53-0
9.077	*Benzyl Acetate*	140-11-4
7.063	*Acetophenone*	98-86-2
7.797	*Nonanal*	124-19-6
9.639	*Decanal*	112-31-2
9.063	*Ethyl Benzoate*	93-89-0

Table S3. Main Volatile Organic Compounds analyzed. Data are given as percentage based on at least three biological replicates. (RT) Retention time.

VOCs:	ZT 0			ZT 3			ZT 6			ZT 9			ZT 12			ZT 15			ZT 18			ZT 21		
	W.T.	GI1	P value	W.T.	GI1	P value	W.T.	GI1	P value	W.T.	GI1	P value .	W.T.	GI1	P value	W.T.	GI1	P value	W.T.	GI1	P value	W.T.	GI1	P value
Mehyil Benzoate	79.9	34.3	1.65E-04	85.8	66.3	3.02E-02	91.3	75.5	1.50E-01	85.7	75.2	1.82E-01	76.8	81.2	2.26E-01	77	77.5	3.01E-01	75.1	74.4	2.64E-01	76.4	67.0	4.13E-01
Benzaldehyde	2.6	0.00	1.18E-06	2.3	0.00	6.85E-04	0.3	0.8	8.77E-01	3.4	3.4	1.96E-01	6.5	8.5	2.66E-01	11.7	5.7	1.94E-01	11.3	4.9	1.97E-01	8.5	1.7	3.97E-02
Isoeugenol	5.0	54.3	7.03E-01	0.3	12.7	1.52E-03	1.7	15.7	1.35E-01	6.1	4.4	1.42E-01	8.7	9.4	2.29E-01	5.0	7.9	4.58E-01	5.7	8.3	3.69E-01	6.0	7.9	8.49E-01
Benzyl Benzoate	4.8	2.1	4.74E-02	4.7	3.7	1.12E-01	1.1	2.0	3.48E-02	2.5	2.3	1.68E-01	5.4	0.5	1.97E-01	3.4	5.6	4.61E-01	3.6	9.2	6.76E-01	3.5	5.2	7.01E-01
Phenylacetaldehyde	0.4	0.00	2.13E-08	0.3	0.00	8.18E-02	0.00	0.3	1.72E-02	0.3	0.3	2.22E-01	1.0	0.1	2.16E-01	1.6	0.9	2.10E-01	1.8	0.8	1.95E-01	1.3	0.4	4.15E-02
Benzyl Alcohol	2.7	0.6	1.41E-04	2.6	3.6	1.46E-01	0.9	0.3	8.95E-02	0.00	0.2	1.74E-02	0.1	0.01	2.29E-01	0.2	0.3	4.56E-01	1.1	1.0	2.47E-01	2.4	1.6	2.87E-01
2.phenylethanol	2.2	6.1	3.43E-05	1.8	3.2	1.83E-02	0.5	0.00	9.03E-02	0.1	0.3	8.32E-01	0.3	0.04	2.59E-01	0.5	0.6	3.63E-01	0.9	1.00	2.59E-01	1.5	1.3	3.90E-01
Methyl Salicylate	0.6	0.2	1.41E-02	0.9	1.6	2.54E-02	1.6	0.9	1.21E-01	1.09	1.05	1.93E-01	0.5	0.06	2.37E-01	0.3	0.3	3.19E-01	0.2	0.4	4.18E-01	0.2	0.8	1.07E-03
Eugenol	2.5	0.07	3.47E-06	0.6	1.3	1.05E-02	0.06	0.00	8.07E-02	0.05	0.12	6.10E-01	0.06	0.00	1.15E-01	0.04	0.1	9.81E-01	0.1	0.00	1.20E-01	0.1	0.4	1.55E-01
Benzyl Acetate	0.6	0.1	2.79E-04	0.6	0.5	9.94E-02	0.2	0.00	6.46E-02	0.00	0.00	1.00E+00	0.00	0.00	3.47E-01	0.03	0.02	2.37E-01	0.1	0.02	2.46E-01	0.00	0.3	9.52E-01
Acetophenone	0.03	0.4	5.00E-02	0.06	0.3	8.13E-01	0.1	0.00	1.39E-01	0.00	0.3	4.69E-02	0.1	0.01	1.85E-01	0.2	0.07	2.14E-01	0.06	0.00	1.39E-01	0.00	0.8	9.23E-02
Nonanal	0.04	0.10	4.34E-01	0.2	0.00	5.36E-03	0.7	2.3	7.75E-02	0.31	10.3	9.31E-01	0.07	0.02	6.94E-01	0.00	0.06	8.62E-02	0.00	0.00	1.00E+00	0.00	0.1	5.74E-02
Decanal	0.00	0.00	1.77E-01	0.08	0.00	8.47E-03	0.4	1.4	9.05E-02	0.00	0.3	3.67E-02	0.04	0.01	7.26E-01	0.00	0.00	1.00E+00	0.00	0.00	1.00E+00	0.00	0.01	3.47E-01
Ethyl Benzoate	0.05	0.6	3.58E-01	0.00	5.3	3.30E-04	0.00	0.6	2.73E-01	0.07	1.8	3.16E-02	0.04	0.2	3.55E-01	0.02	0.9	3.37E-01	0.00	0.00	1.00E+00	0.00	12.6	3.14E-01

Table S4. Percentage of the main Volatile Organic Compounds analyzed during 24 hours between wild type and the silenced *PhGI1* flowers of T1 generation. P values ≤ 0.05 according to Students T-test were considered as significant.

**PhGi1 For**	TGGAGAAAGGGCAGAGACAT
**PhGi1 Rev**	GTGGAGCCACCCTTACGTT
**PhGi1attb1 For**	GGGGACAAGTTTGTACAAAAAAGCAGGCTTGGAGAAAGGGCAGAGACAT
**PhGi1attb2 Rev**	GGGGACCACTTTGTACAAGAAAGCTGGGTAGTGGAGCCACCCTTACGTT
**SecpDON201 For**	TCGCGTTAACGCTAGCATGGATCTC
**SecpDON201 Rev**	GTAACATCAGAGATTTTGAGACAC
**nptII For**	CCTGCTTGCCGAATATCATGGTGG
**nptII Rev**	CGAAATCTCGTGATGGCAGGTTGG
**Agri 51**	CAACCACGTCTTCAAAGCAA
**Agri 56**	CTGGGGTACCGAATTCCTC
**Agri 64**	CTTGCGCTGCAGTTATCATC
**Agri 69**	AGGCGTCTCGCATATCTCAT
**PhAct For**	TGCACTCCCACATGCTATCCT
**PhAct Rev**	TCAGCCGAAGTGGTGAAAGAG
**PaxilGI1 For**	TTGTACGTGCACTCAGCACA
**PaxilGI1 Rev**	CCATTTTTGATTCGGCTGTT
**PhGi2 For**	TTTAGAGTCCTTTCACTCATCCATC
**PhGi2 Rev**	AATACAGCATTTGTTACATGGAGGT
**PhZTL For**	TGCATCTGTTGGCTCTGTTT
**PhZTL Rev**	CCCCAACCCAATCTCTTAGC
**PhLHY For**	CGACGTGGTAGGAATTGCATC
**PhLHY Rev**	GACCGAAATGGTCATCAAAGGAC
**PhTOC1 For**	TGATGGTAAGGGGAGCAAAG
**PhTOC1 Rev**	CTGAAGCAGGATGCCCATTA
**PhELF4 For**	GCCACCACCAAACCCTAAC
**PhELF4 Rev**	AGGCTTCAAAGGCAGTGCT

Table S5. List of primers used.

```
PhGI1        GAAAGCAGAAAGGCAAAGGATCTAAGAAAGTGAAAAAACAAGGACCTGTTTCAGCGTTTG      60
PhGI2        AAAAACCGAAACAGCTGGGAAGTAAAAAGGTGAAGAAACAAGGGCCCGTTGCAGCATTTG      60
FN036363     ------------------------------------------------------------      0


PhGI1        ATTCTTATGTTCTTGCTGCTGTCTGTGCTCTTTCATGTGAACTTCAACTCTTTCCTTTGA      120
PhGI2        ATTCTTACGTACTTGCTGCTGTGTGTGCTCTTTCTTGTGAACTTCAGCTCTTCACTTTAA      120
FN036363     ------------TTGCTGCTGTGTGTGCTCTTTCTTGTGAACTTCAGCTCTTCACTTTAA      48
                         ********** ***********:***********.***** .****.*

PhGI1        TTTCAAGAGGGACTAATTATTCAGATCCAAAAACCATCCCCGATGCTGCAAAGCATGCAA      180
PhGI2        TTTCAAGAGGGACTAATCATCCAGATCCAAAAATCATCATGGATGAAGCAAAGCCCGCAA      180
FN036363     TTTCAAGAGGGACTAATCATTCAGATCCAAAAATCATCATGGATGAAGCAAAGCCCGCAA      108
             ***************** ** ************ ****.  ****.:*******. ****

PhGI1        ATGATTCGTCTATTGAGTTAAAAAATGGGATGCATTCTGCTATCTATCATACTCGGAGGA      240
PhGI2        ATGATTCGTCGATTGAGTTACGAAACGGGATTCATTCTGCAGTCTGTCATACTCGGAGAA      240
FN036363     ATGATTCGTCGATTGAGTTACGAAACGGGATTCATTCTGCCGTCCGTCATACTCGGAGAA      168
             ********** *********..*** ***** ******** .** .************.*

PhGI1        TATTGGCAATTCTGGAGGCTCTTTTCTCTTTGAAACCATCATCTGTTGGAACTTCATGGA      300
PhGI2        TATTAGCAATTTTAGAGGCTCTTTTTTCTTTGAAACCATCATCTGTTGGAACCTCATGGA      300
FN036363     TATTAGCAATTTTAGAGGCTCTTTTTTCTTTGAAACCATCATCTGTTGGAACCTCATGGA      228
             ****.****** *.*********** ************************** *******

PhGI1        GCTACAGCTCAAATGAGATAGTTGCTGCAGCTATGGTAGCTGCTCATATATCTGATTTGT      360
PhGI2        GCTACAGCTCAAATGAGATAGTTGCTGCAGCTATGGTAGCTGCTCATATTTCTGATCTGT      360
FN036363     GCTACAGCTCAAATGAGATAGTTGCTGCAGCTATGGTAGCTGCTCATATTTCTGATCTGT      288
             *************************************************:****** ***

PhGI1        TTAGATGCTCCAAGGCTTGCATGCATTCTCTTTCAATCTTAATAAGGTGTAAGTGGGATA      420
PhGI2        TTAGACGCTCCAAGGCTTGCATGCAGGGTCTTGCTATCTTGATACGGTGTAAGTGGGATA      420
FN036363     TTAGACGCTCCAAGGCTTGCATGCAGGGTCTTGCTATCTTGATACGGTGTAAGTGGGATA      348
             ***** *******************   **** *:*****.***.***************

PhGI1        ATGAAATTCACTCCAGAGCATCTTCGCTGTATAACCTCATTGACATTCATAGCAAAGTTG      480
PhGI2        ATGAAATTCATTCAAGGGCATCTTCACTCTATAACCTAATTGATATTCATAGTAAGACTG      480
FN036363     ATGAAATTCATTCAAGGGCATCTTCACTCTATAACCTAATTGATATTCATAGTAAGACTG      408
             ********** **.**.********.** ********.***** ******** **.. **

PhGI1        TTGCATCAATTGTTGACAAGGCCGAACCATTGGAAGCACACCTAATACCTGCACCTGTTC      540
PhGI2        TTGCATCAATTGTCAACAAGGCTGAACCATTGGAAGCTTATTTAATACATGCACCAATTC      540
FN036363     TTGCATCAATTGTCAACAAGGCTGAACCATTGGAAGCTTATTTAATACATGCACCAATTC      468
             ************* .******* **************: *  ******.******:.***

PhGI1        TTAATAAAAGAAGCATGTGCGTGAATGGGAGAAAACGTAATAAATATAGCACTTGTAGCT      600
PhGI2        TGAAGGAGCGGCCCAGGTGCTTAAATGGGAAGAAACATTATAAATATACCAATCGTAACT      600
FN036363     TGAAGGAGCGGCCCAGGTGCTTAAATGGGAAGAAACATTATAAATATACCAATCGTAACT      528
             * ** .*..*.. ** **** *.*******..****.*:********* **.* ***.**

PhGI1        GCTTAACTGCAGAACAATCATCGTTGCTCGAGTGCAACTCAACTGATTGTAAAAGTTTAA      660
PhGI2        GCTTAACAGCAGAAGAGCCATCAGTGTCACAGTGTAAAGATTC------------CTCTA      648
FN036363     GCTTAACAGCAGAAGAGCCATCAGTGTCTCAGTGTAAAGAT-------------------      569
             *******:****** *. ****. **    **** **. .:

PhGI1        TTATGAGTGAGAAAGTTTTACACTCAAGTGAGGTTTCACAGTGCGCCACTGGTAAAGGGA      720
PhGI2        ATTGGTGCGACAAAGCTTCACACCCAAGTGAGGTTGCTGGAGGCACCATCAGCAAAGTGT      708
FN036363     ------------------------------------------------------------      569
```

Figure S1. DNA alignment of *PhGI1* (Peaxi132Scf1428Ctg026), *PhGI2* (Peaxi132Scf1428Ctg060) from *Petunia hybrida* and the *PhGI* sequence FN03636 reported by Fenske et al. (2015) [24], using Clustal Omega (https://www.ebi.ac.uk/Tools/msa/clustalo/).

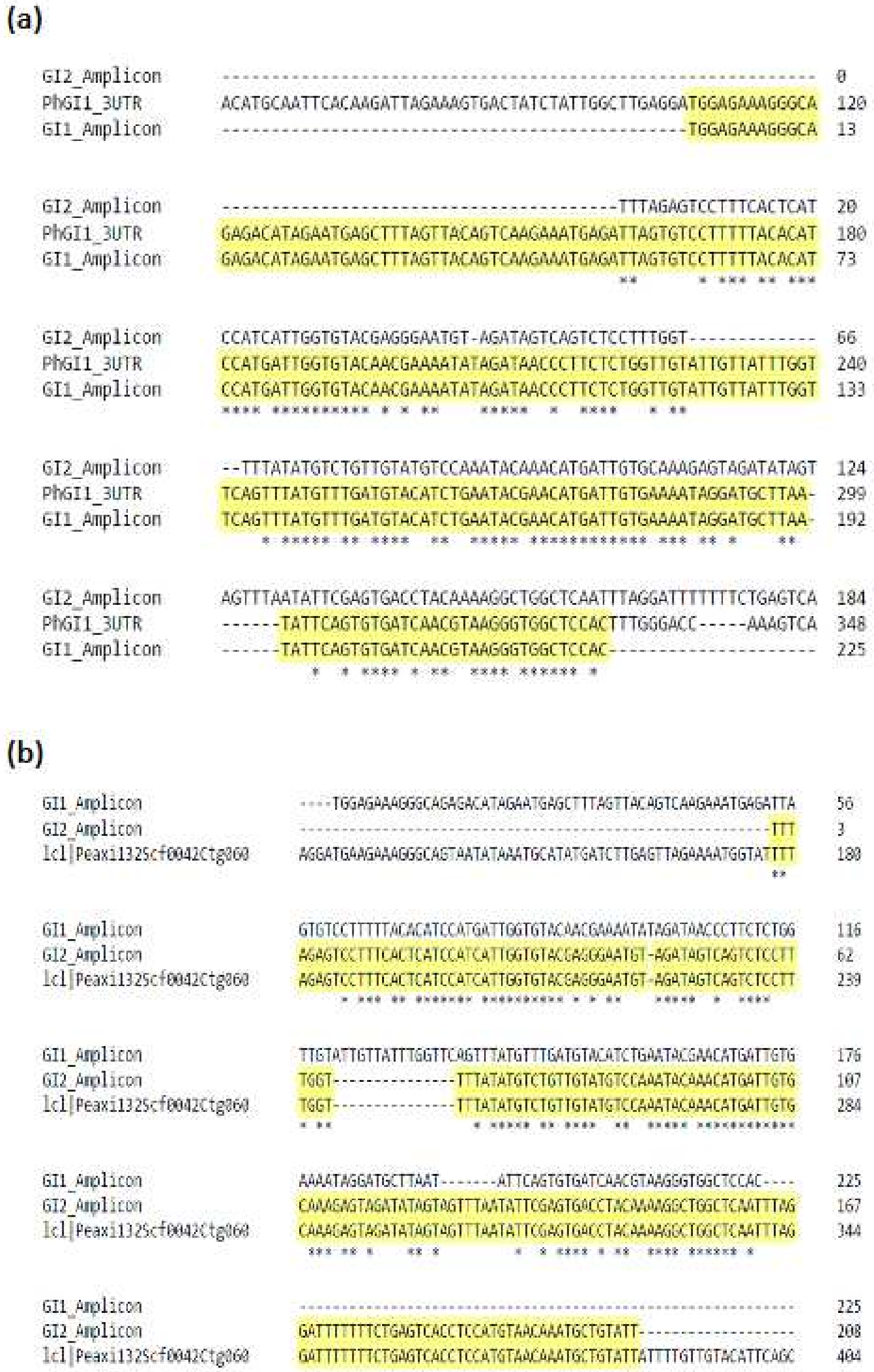

Figure S2. **Figure S2.** Alignment between (a) amplified regions of *PhGI1* and *PhGI2 (GI1_Amplicon and GI2_Amplicon)* and the 3'UTR region of *PhGI1* sequence from *Petunia hybrida (PhGI1_3'UTR)* and (b) amplified regions of *PhGI1* and *PhGI2 (GI1_Amplicon and GI2_Amplicon)* and *PhGi2* sequence from *Petunia axillaris* (Peaxi132Scf1428Ctg060) using Clustal Omega (https://www.ebi.ac.uk/Tools/msa/clustalo/). Regions marked in color emphasize on sequence similarity between amplicons and genomic clones.

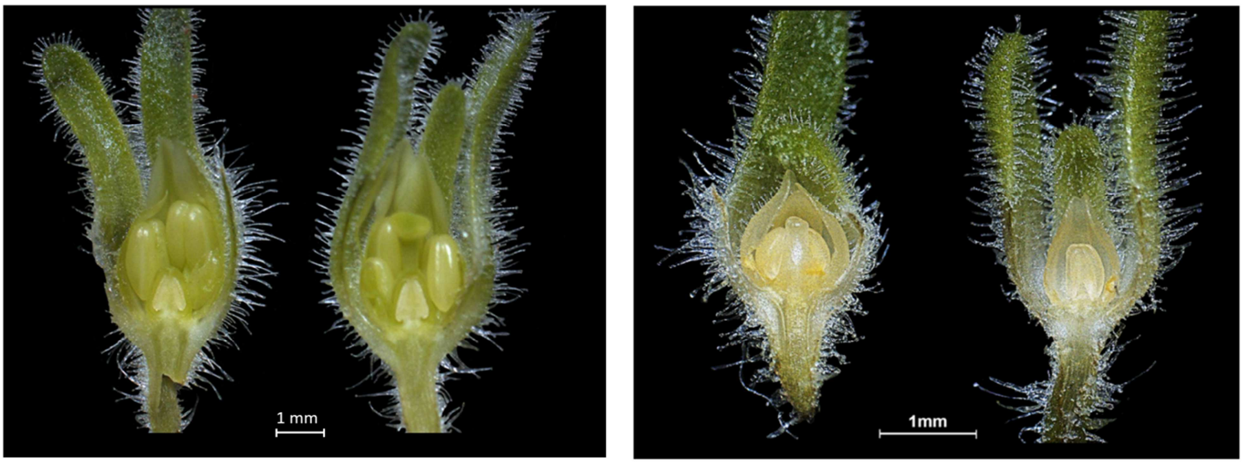

Figure S3. Fully developed flower bud (left) of a wildtype line compared to aborted flower bud (right) of *iRNA::PhGI1* T1 line 3.7 as observed in all *iRNA::PhGI* lines.

(a)

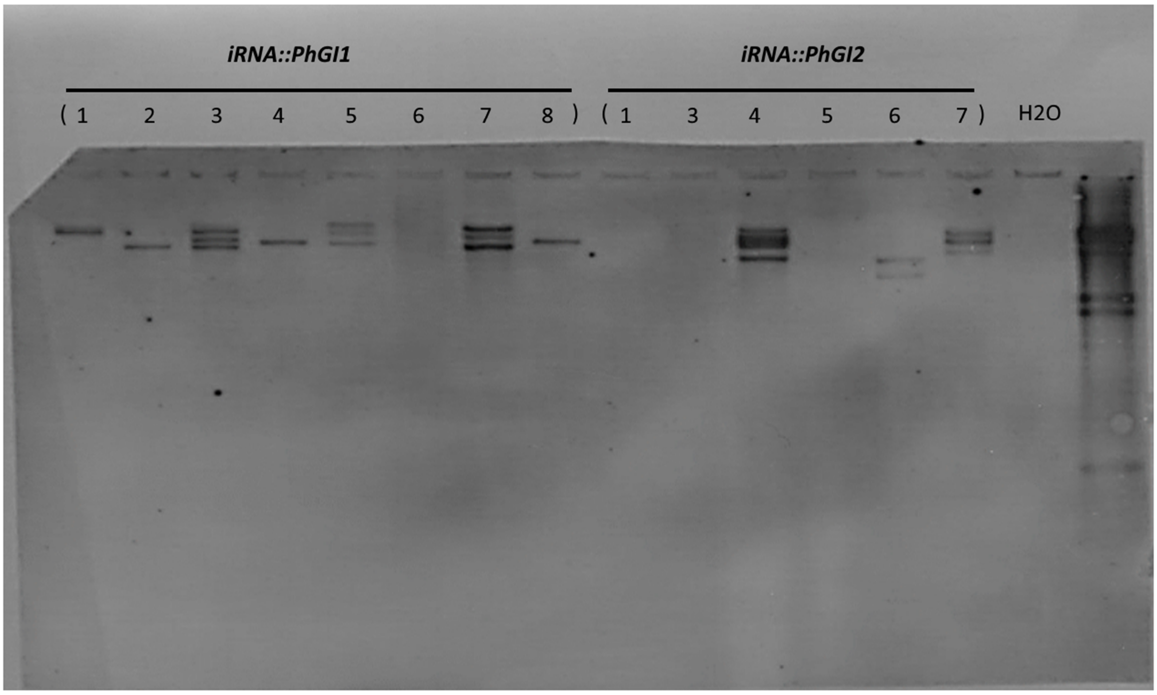

(b)

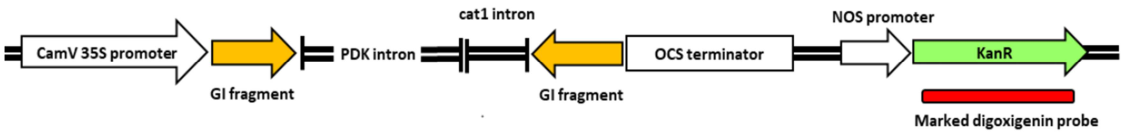

Figure S4. Southern Blot analysis of the T0 generation. (a) Result of T0 lines of *iRNA::PhGI1* on the left and *iRNA::PhGI2* on the right. (b) Schematic representation of the localization of the marked digoxigenin probe used.
